# Assessing Dynamic Changes of Taste-Related Primary Metabolism During Ripening of Durian Pulp Using Metabolomic and Transcriptomic Analyses

**DOI:** 10.3389/fpls.2021.687799

**Published:** 2021-06-18

**Authors:** Lalida Sangpong, Gholamreza Khaksar, Pinnapat Pinsorn, Akira Oikawa, Ryosuke Sasaki, Alexander Erban, Mutsumi Watanabe, Karan Wangpaiboon, Takayuki Tohge, Joachim Kopka, Rainer Hoefgen, Kazuki Saito, Supaart Sirikantaramas

**Affiliations:** ^1^Molecular Crop Research Unit, Department of Biochemistry, Faculty of Science, Chulalongkorn University, Bangkok, Thailand; ^2^Faculty of Agriculture, Yamagata University, Yamagata, Japan; ^3^Metabolomics Research Group, RIKEN Center for Sustainable Resource Science, Yokohama, Japan; ^4^Max Planck Institute of Molecular Plant Physiology, Potsdam, Germany; ^5^Plant Secondary Metabolism, Graduate School of Science and Technology, Nara Institute of Science and Technology (NAIST), Nara, Japan; ^6^Molecular Sensory Science Center, Chulalongkorn University, Bangkok, Thailand

**Keywords:** *Durio zibethinus*, metabolome analysis, ripening-associated metabolites, taste precursors, transcriptome analysis

## Abstract

Durian is an economically important fruit of Southeast Asia. There is, however, a lack of in-depth information on the alteration of its metabolic networks during ripening. Here, we annotated 94 ripening-associated metabolites from the pulp of durian cv. Monthong fruit at unripe and ripe stages, using capillary electrophoresis- and gas chromatography- time-of-flight mass spectrometry, specifically focusing on taste-related metabolites. During ripening, sucrose content increased. Change in raffinose-family oligosaccharides are reported herein for the first time. The malate and succinate contents increased, while those of citrate, an abundant organic acid, were unchanged. Notably, most amino acids increased, including isoleucine, leucine, and valine, whereas aspartate decreased, and glutamate was unchanged. Furthermore, transcriptomic analysis was performed to analyze the dynamic changes in sugar metabolism, glycolysis, TCA cycle, and amino acid pathways to identify key candidate genes. Taken together, our results elucidate the fundamental taste-related metabolism of durian, which can be exploited to develop durian metabolic and genetic markers in the future.

## Introduction

Durian (*Durio zibethinus* L.) is a highly economically valuable fruit endemic to Southeast Asia, which has recently begun to be distributed globally. Owing to the increasing demand for durian, the price and production of the fruit tend to increase every year. Durian is well known for its outstanding flavor, described as an overpowering sweet taste, with a sweet, fruity odor resulting from high contents of starch, sugar, and saturated fatty acids in the ripe pulp (Charoenkiatkul et al., 2018). More interestingly, ripe durian pulp contains various bioactive compounds, including carotenoids, flavonoids, and polyphenols, which confer substantial antioxidant properties (Aziz and Jalil, 2019), suggesting that consumption of durian may have potential health benefits. The ripening process contributes to the organoleptic properties of durian pulp.

Fruit ripening is a complex and coordinated developmental process associated with pronounced molecular and biochemical changes. The climacteric fruits, including durians, are usually harvested at the commercial maturity stage. Afterward, the fruits undergo a postharvest ripening process, during which the rates of respiration and ethylene production increase dramatically. During this process, stored carbohydrates are broken down to sugars, and acidity is reduced alongside the increase in taste and aroma volatiles (Gao et al., 2020). Moreover, modifications of amino acids and organic acids are linked with fruit aroma by acting as precursors for the biosynthesis of aroma-forming volatile compounds (Tieman et al., 2006). Taken together, these modifications are the key contributors to fruit flavor.

Over the past decade, the advent of “omics” approaches has significantly contributed to identifying functional metabolites in primary and secondary plant metabolism during fruit development. Metabolomics has been successfully used to identify primary metabolites, including sugars, organic acids, amino acids, and other related compounds, and to provide an understanding of the whole landscape of metabolic alteration during the development and ripening of fruits, such as peach, tomato, strawberry, and grape (Lombardo et al., 2011). Moreover, metabolomics, coupled with transcriptomics, provides information on major metabolic networks and candidate genes controlling the underlying processes; integration of these data with genomics provides new insights into the major metabolic variations and their genetic and biochemical control during development. Such studies have widely been performed on tomato, a popular model fruit, and documented how breeding and genetic selection globally altered tomato fruit metabolite content (Zhu et al., 2018).

Metabolic profiles of ripe durian pulp have been investigated using capillary electrophoresis-time of flight/mass spectrometry (CE-TOF/MS) (Pinsorn et al., 2018). Cultivar-dependent metabolites associated with the sensory traits of durian fruit pulp, such as its odor-related (cysteine and leucine) and ripening-associated (aminocyclopropane carboxylate) metabolites, are of utmost importance. In 2017, the draft genome of *Durian zibethinus* was first published, and genome data integrated with transcriptome data identified methionine-γ-lyase as a key gene involved in controlling sulfur volatile compound production in durian pulp (Teh et al., 2017). This result was consistent with previously identified odor-active compounds in durian pulp, such as methanethiol (Li et al., 2012). Furthermore, volatile-aroma esters, such as ethyl (2S)-2-methylbutanoate and ethyl butanoate, were isolated in the study, but their related pathways have not been studied in durian. The genomic data led to further studies to better understand the durian ripening process. Subsequently, a genome-wide analysis of the Dof (DNA binding with one finger) transcription factor family identified 24 Dofs (*DzDofs*), among which 15 were expressed in the fruit pulp. Functional characterization of *DzDof2.2* suggested that it exerts its effects on fruit ripening by regulating auxin biosynthesis and auxin-ethylene crosstalk (Khaksar et al., 2019). In addition, a member of the auxin response factor (ARF) transcription factor family was identified, showing that DzARF2A mediates durian fruit ripening through transcriptional regulation of ethylene biosynthesis genes (Khaksar and Sirikantaramas, 2020). In this regard, omics analyses can be powerful tools to improve our limited understanding of the mechanisms underlying the durian postharvest ripening process, especially the changes in primary metabolites. In this study, we performed metabolomics, including capillary electrophoresis (CE) and gas chromatography (GC) coupled with time-of-flight mass spectrometry (TOF/MS), and transcriptomics to elucidate the post-harvest ripening-associated metabolic processes of the Monthong cultivar, the most widely cultivated durian in Thailand. Our findings provide comprehensive information on metabolic shifts during these stages. The identification of ripening-associated metabolites, which contribute to its unique flavors, and key candidate genes may be further exploited in durian breeding programs to develop cultivars with altered sensory characteristics or enhanced nutritional value.

## Materials and Methods

### Plant Materials and Sample Preparation

Durian cv. Monthong fruits were harvested with at least three replicates of each stage from an orchard in the Trat province of Thailand in 2016. The three stages of the durian fruit used in this study are as follows: unripe, midripe, and ripe. For the unripe stage, the fruit was harvested at the commercially mature stage of 105 days after anthesis. The fruit at midripe and ripe stages were harvested at the commercially mature stage and kept at room temperature (28 °C) for postharvest ripening until reaching a firmness of 3.4 ± 0.81 N (∼3 days after harvest) and 1.55 ± 0.45 N (∼5 days after harvest), respectively (Khaksar et al., 2019). At each stage, durian pulp was collected from the central seed of each locule, immediately frozen in liquid nitrogen, and ground into powder. The powder was either freeze-dried for CE-TOF/MS, GS-TOF/MS, and high-performance anion-exchange chromatography with pulsed amperometric detection (HPAEC-PAD) analyses or stored at −80°C for RNA extraction. Another seed from a different locule of the same fruit was used to analyze firmness, using a texture analyzer, to ensure that samples of the same stage from different cultivars were under identical conditions.

### Determination of Total Soluble Solids (TSS) and Titratable Acidity

Two sets of a freeze-dried sample (20 mg, at least three replicates) were homogenized in 200 μL of distilled water. The homogenate was centrifuged at 14,000 × *g* at 18 C for 20 min. The supernatant from the first set was used to measure total soluble solids (TSS) using a digital refractometer (Hanna Instruments, Woonsocket, RI, United States). The other set of the supernatants was used to quantify titratable acidity modified from Tan et al. (2019). Briefly, the supernatant was titrated against 0.1 M NaOH mixed with 2 μL of phenolphthalein until the solution turned pink. Titratable acidity was calculated using 1 mg malic acid/100 mg dried sample, where the milliequivalent factor of malic acid is 0.067. The formula is as follows:

$$\begin{array}{c} \%\mathrm{acid}=\frac{\begin{array}{c} \mathrm{NaOH}\mathrm{used}\left(\mu L\right)\times \mathrm{molarity}\mathrm{of}\mathrm{NaOH}\mathrm{used}\\ \times \left(\mathrm{miliequivalent}\mathrm{factor}\right)\times 100 \end{array}}{\begin{array}{c} \mathrm{Amount}\mathrm{of}\mathrm{sample}\mathrm{used}\left(\mathrm{mg}\right) \end{array}} \end{array}$$

### Metabolite Analysis Using CE-TOF/MS

Metabolite extraction was performed according to our previous method (Pinsorn et al., 2018). In brief, the freeze-dried sample (5 mg, five replicates) was mixed with methionine sulfone and camphor 10-sulfonic acid as internal standards (Sigma-Aldrich, St. Louis, MO, United States). CE-TOF/MS analysis was then performed as previously described (Oikawa et al., 2011). Methionine sulfone and camphor 10-sulfonic acid were used for cation and anion analyses, respectively, and their peak areas were used to normalize the peak areas of metabolites, providing the relative intensity of each metabolite. In addition, the CE-TOF/MS data of the ripe-stage durian harvested in 2016 were retrieved from our previous paper (Pinsorn et al., 2018).

### Metabolite Analysis Using GC-TOF/MS

Metabolite extraction, derivatization, and chromatography data processing were performed according to the methods described previously (Erban et al., 2020). In brief, the freeze-dried sample (10 mg, three replicates) was mixed with 360 μL methanol-mix that includes ^13^C_6_-sorbitol as an internal standard, followed by 200 μL chloroform and 400 μL water that are added for phase separation. Solvents were of highest available purity (Merck, Darmstadt, Germany). Samples were vortexed and agitated 15 min at 70 °C after adding the methanol-mix, incubated 5 min at 37 °C after chloroform addition, and thoroughly vortexed after water addition. Phase separation was induced by centrifugation. The upper polar phase (80 μL) was dried. Each sample was chemically derivatized using methoxyamine hydrochloride in pyridine and BSTFA (Macherey-Nagel, Düren, Germany) as previously described (Erban et al., 2020), including *n*-alkanes for retention index calculation. The derivatized samples (1 μL) were analyzed by splitless and 1:30 split-injection modes with a 6890N gas chromatograph (Agilent, Santa Clara, CA, United States) connected to a Pegasus III time-of-flight mass spectrometer (Leco Instruments, St. Joseph, MI, United States). Metabolite annotation was performed by matching mass spectra and retention index information to the Golm Metabolome Database using TagFinder software (Kopka et al., 2005; Luedemann et al., 2008; Erban et al., 2020).

### Measurement of Sugar Content Using HPAEC-PAD Analysis

A freeze-dried sample (15 mg, at least three replicates) was extracted in 1 mL of 80% methanol, with 0.3 mg of cellobiose (Wako, Osaka, Japan) as an internal standard for calibration, and incubated at 92 °C for 10 min. The extraction step was repeated twice. The supernatant was transferred to a new tube and evaporated with CentriVap Centrifugal Vacuum Concentrators. The pellet was dissolved in 300 μL of ultrapure (UP) water, 50-fold diluted, and filtered through a 0.22-μm syringe filter for further analysis.

For HPAEC-PAD, Dionex ICS 5000 ion chromatography system (Dionex, Sunnyvale, CA, United States) was used with a Carbo PacTM PA1 high-performance anion-exchange column (4 × 250 mm) (Dionex). The elution buffer system was consisted of 150 mM NaOH (buffer A) and 500 mM CH_3_COONa (Kemaus, Cherrybrook, Australia) in 150 mM NaOH (buffer B). The column was equilibrated by buffer A with a flow rate of 1 mL/min at 30 C. The samples were eluted by multistep gradients as follows: constant 100% buffer A at 0–5 min, linear gradient to 5% buffer B at 5–8 min and constant at 5% buffer B to 12 min, linear gradient to 20% buffer B at 12–15 min, linear gradient to 100% buffer B at 15–17 min and hold at 100% buffer B for 2 min, and linear gradient to 100% buffer A at 19–20 min.

Quantification of the targeted sugars, including *myo*-inositol (Phytotechnology Laboratories, Lenexa, KS, United States), galactinol (TCI, Tokyo, Japan), sorbitol (Acros, Antwerp, Belgium), fructose (Sigma), glucose (Univar, Downers Grove, United States), 1-kestose (Wako), maltose (Condalab, Madrid, Spain), raffinose (TCI), and sucrose (Univar), was conducted using mixtures of the reference standards in the ranges of 0.1–2.5 mg/mL.

### RNA Extraction and Transcriptome Analysis

Total RNA was extracted (in triplicate) from the samples at unripe and ripe stages, using the PureLink Plant RNA Reagent (Invitrogen Carlsbad, CA, United States) per the manufacturer’s instructions. cDNA was synthesized using the RevertAid First Strand cDNA Synthesis Kit (Thermo Fisher Scientific Inc., Waltham, MA, United States) according to the manufacturer’s instructions. Next, transcriptome sequencing was performed using the BGISEQ-500 platform (BGI, Shenzhen, China). We used the OmicsBox program (v1.4.1.1) for transcriptome data analysis. Filtering of raw reads was performed, which resulted in paired-end clean reads, and unpaired reads that lost their corresponding sequence partners due to quality control procedures. Then, the *de novo* transcriptome assembly of the cleaned reads was performed using the Trinity (v2.8.5) package of the program with default parameters. The assembled transcripts were identified ORF and annotated with NCBI BLAST against non-redundant protein sequences (Nr v5), InterProScan, Gene Ontology (GO), and Kyoto Encyclopedia of Genes and Genomes (KEGG). The clean reads were mapped back to the assembled transcripts using the Bowtie2 (v2.3.5.1) package with default parameters, and the expression levels of the transcripts were quantified using the RSEM (v1.3.1) package with default parameters. The read counts were normalized using the reads per kilobase of transcript and per million mapped reads (RPKM) method. Pairwise differential expression analysis was performed using the exact test (FDR < 0.05) of the software package edgeR (v3.28.0) when change in expression was two-fold or higher. The differentially expressed genes (DEGs) were classified using GO classification and into specific biological pathways using KEGG.

### Reverse Transcription Quantitative Polymerase Chain Reaction (RT-qPCR) Analysis

cDNA was used for gene expression analysis with RT-qPCR; the reaction mix was prepared using the Luna® Universal qPCR Master Mix (New England Biolabs, Ipswich, MA, United States) according to the manufacturer’s protocol and the reaction was run on a CFX Real-Time PCR system (Bio-Rad Laboratories, Inc., Hercules, MA, United States). Relative expression levels were calculated using the 2^–ΔΔCT^ method (Livak and Schmittgen, 2001), based on the ratio of the expression of candidate genes and elongation factor 1 alpha (*EF1a*), a housekeeping gene (Khaksar and Sirikantaramas, 2020). The primers used for RTqPCR analyses are listed in Supplementary Table 1.

### Statistical Analysis

The metabolite data were processed by algorithms in MetaboAnalyst 5.0^1^ (Pang et al., 2021). Principal component analysis (PCA), which is a dimension-reduction tool, was applied to investigate the relationships between samples. A volcano plot was used to select significant metabolites, which were designated as those with a relative intensity greater than the threshold (| log_2_(fold change [FC])| > 1, *P* < 0.05) between the two stages. Metabolomics Pathway Analysis (MetPA) was performed to enrich the metabolite set into the relevant pathways. Pathway impact of each metabolic pathway is calculated from the sum of the importance measures of the matched metabolites divided by the su of the importance measures of total metabolites. Pathways that reached cut-off values (*P* < 0.05, pathway impact ≥ 0.1) were considered perturbed (Guo and Tao, 2018). One-way ANOVA followed by Tukey’s test in GraphPad Prism version 5 was used to compare the means of each sugar at different ripening stages (*P* < 0.05).

## Results and Discussion

### An Overview of Metabolites Involved in Durian Fruit Ripening

**FIGURE 1:**
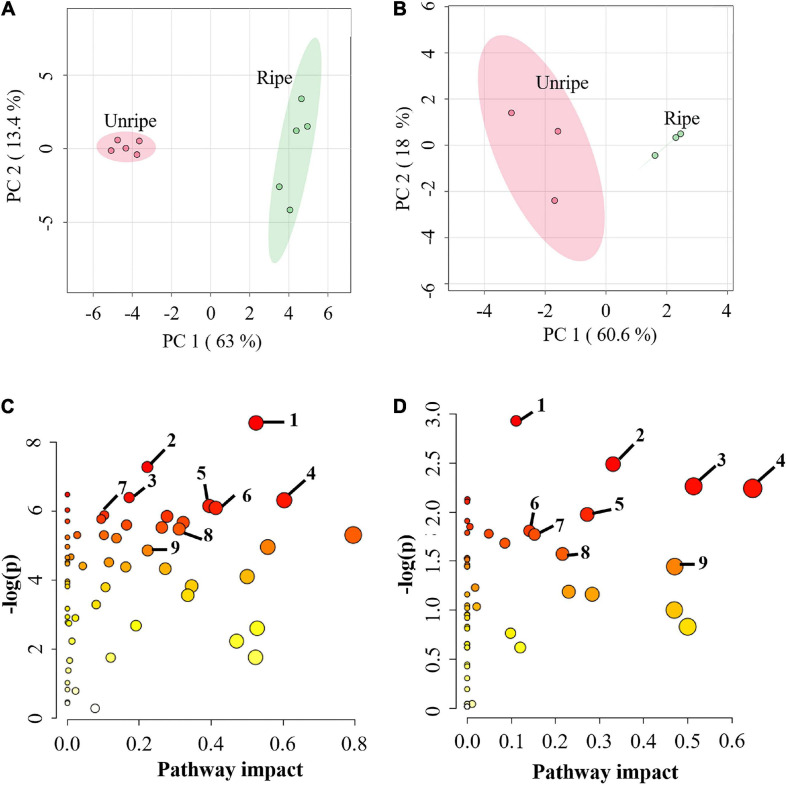
**(A)** Principal component analysis (PCA) score plot of metabolites from capillary electrophoresis-time of flight/mass spectrometry (CE-TOF/MS) and **(B)** from gas chromatography- time-of-flight mass spectrometry (GC-TOF/MS) in the pulp of durian cv. Monthong at unripe and ripe stages, which are labeled in red and green, respectively. Each dot represents each biological replicate sample, and the circle represents 95% confidence intervals. **(C)** Metabolic pathway analysis (MetPA) of metabolites from CE-TOF/MS and **(D)** from GC-TOF/MS. Matched pathways related to ripening of durian cv. Monthong are displayed as circles. The color and size of the circles represent the *P* value and pathway impact value, respectively. The significantly altered pathways associated with our detected primary metabolites were labeled. Metabolic pathways with pathway impact > 0.1 and *P* < 0.05 were perturbed pathways. The top 7 perturbed pathways of **(C)** are as follows: 1, arginine and proline metabolism; 2, aminoacyl-tRNA biosynthesis; 3, glutathione metabolism; 4, glycine, serine and threonine metabolism; 5, cysteine and methionine metabolism; 6, glyoxylate and dicarboxylate metabolism; 7, vitamin B6 metabolism. The central carbon pathways of **(C)** are: 8, Citrate cycle (TCA cycle); 9, glycolysis. All perturbed pathways of **(D)** are: 1, aminoacyl-tRNA biosynthesis; 2, starch and sucrose metabolism; 3, glycine, serine and threonine metabolism; 4, alanine, aspartate and glutamate metabolism; 5, galactose metabolism; 6, glutathione metabolism; 7, arginine and proline metabolism; 8, tyrosine metabolism; 9, phenylalanine metabolism.

Durian pulp undergoes physiological changes during ripening, including slight yellowing of the pulp, during postharvest ripening (Supplementary Figure 1). The total soluble solid (TSS) level of durian pulp increased significantly from the unripe stage (Supplementary Figure 2A). The accumulation of TSS increases consistently with total sugar content but is negatively related to starch content (Ketsa and Daengkanit, 1998) relative to the sweetness of the ripe pulp. At the same time, the percentage of titratable acidity (%TA) did not change significantly during ripening. However, %TA tended to increase during durian ripening (Supplementary Figure 2B). Titratable acidity refers to total acid contents inside the food (Sadler and Murphy, 2010), suggesting that the acidity of durian pulp does not change considerably as it ripens. The changes in the underlying mechanisms during the ripening process contribute to the durian fruit’s characteristics. To study the alterations of primary metabolites in these related mechanisms, we performed metabolome analysis in durian cv. Monthong pulp at the unripe and ripe stages. CE-TOF/MS and GC-TOF/MS techniques were used for comprehensive analysis of primary metabolites, which identified 167 and 56 metabolites, respectively (Supplementary Table 2). Metabolites were further analyzed using the web-based tool MetaboAnalyst 5.0. PCA plots, showed the total variance of the CE-TOF/MS data was 63% from PC1 and 13.4% from PC2 (Figure 1A), while total variance of the GC-TOF/MS data was 60.6% from PC1 and 18% from PC2 (Figure 1B). According to these two PCA plots, unripe durian samples were clearly separated from the ripe groups in PC1. As expected, the results showed that ripening greatly influences metabolic changes at the biochemical level and leads to physiological changes, such as the increased flavor and softening of the edible ripe fruit. This phenomenon is likewise observed in other fruit species, such as peach strawberry, tomato, and grape (Carrari and Fernie, 2006; Lombardo et al., 2011), which each constitute different fruit models. To obtain the relevant biological pathways, Metabolomic Pathway Analysis (MetPA) was used. CE-TOF/MS results indicated that of 59 enriched biological pathways identified by KEGG analysis, 29 metabolic pathways were perturbed (Supplementary Table 3), including central carbon pathways (glycolysis and TCA cycle) and several amino acid pathways (Figure 1C). Meanwhile, GC-TOF/MS results indicated that nine pathways were significantly impacted (Figure 1D), including starch and sugar metabolism, as well as some amino acid pathways that were previously found in the CE-TOF/MS analysis (Supplementary Table 3). These results demonstrated that primary metabolic pathways were significantly altered during durian ripening, which may involve in flavor precursor biosynthesis.

As observed, there was some overlap in pathways identified by each method. We further investigated metabolites in those pathways. Among these metabolites, 28 were annotated using both techniques (Supplementary Table 2 and Supplementary Figure 3). In total, 195 annotated metabolites were obtained and used for further analysis. Our annotation showed that results from each technique validated those from other techniques because the same metabolites exhibited similar changes in intensity during the ripening period (Supplementary Table 2). It is known that both CE-TOF/MS and GC-TOF/MS annotate slightly different kinds of primary metabolites. CE-TOF/MS can separate a wider range of charged metabolites, including amino acids and organic acids, while GC-TOF/MS is more dependent on volatility and can identify uncharged metabolites, such as sugar and sugar derivatives.

### Ripening-Associated Metabolites Contributing to Durian Flavor

**FIGURE 2:**
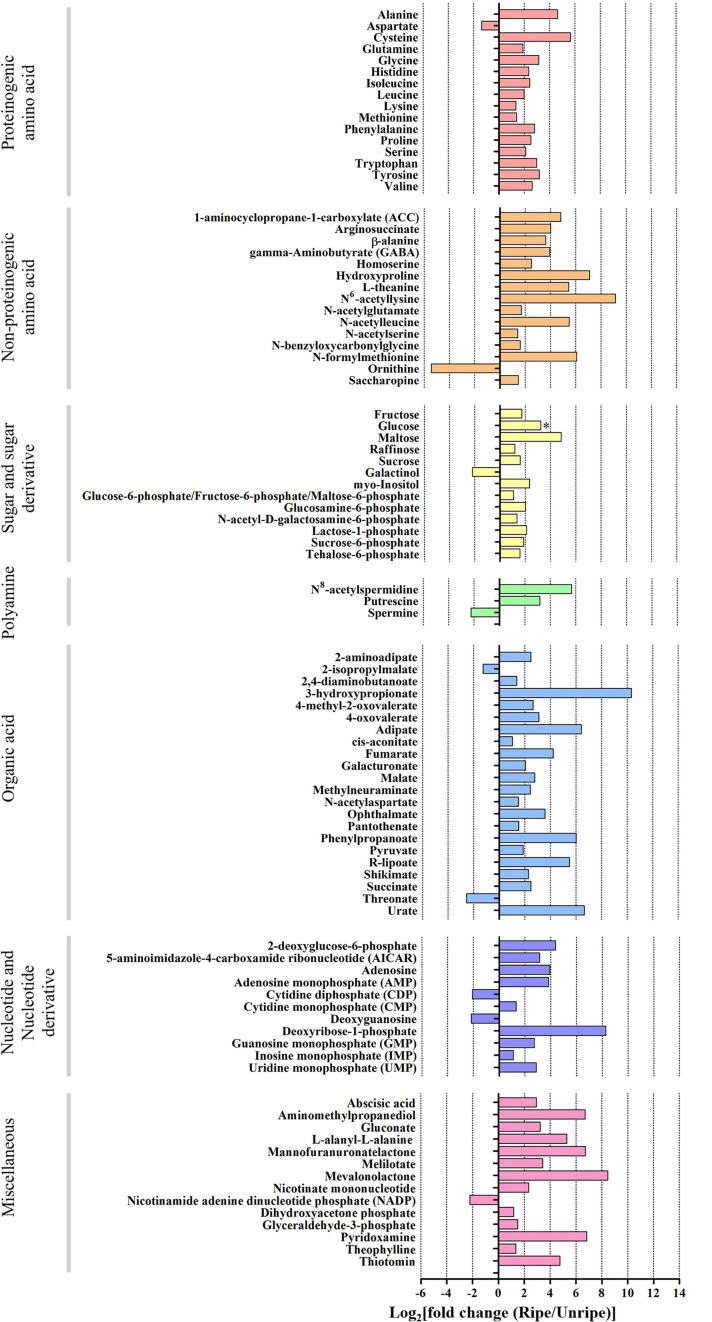
Log_2_ of fold change (FC) of metabolite intensity from ripe and unripe stages of durian cv. Monthong. Based on volcano plot (| log_2_(FC)| > 1, *P* < 0.05), these metabolites significantly changed during the ripening process. Changes are represented in the bar chart, indicating increased levels (positive values) or decreased levels (negative values) of the metabolite intensity. (^∗^ = glucose which is significant under *P* = 0.1).

Out of 195 annotated metabolites, 94 in durian pulp were significantly altered between the unripe and ripe stages (Figure 2, Supplementary Figure 4, and Supplementary Table 4). The ripening-associated metabolites were classified into six groups: proteinogenic amino acids, non-proteinogenic amino acids, sugar and sugar derivatives, polyamines, organic acids, and nucleotide and nucleotide derivatives. All others were placed in a miscellaneous group.

**FIGURE 3:**
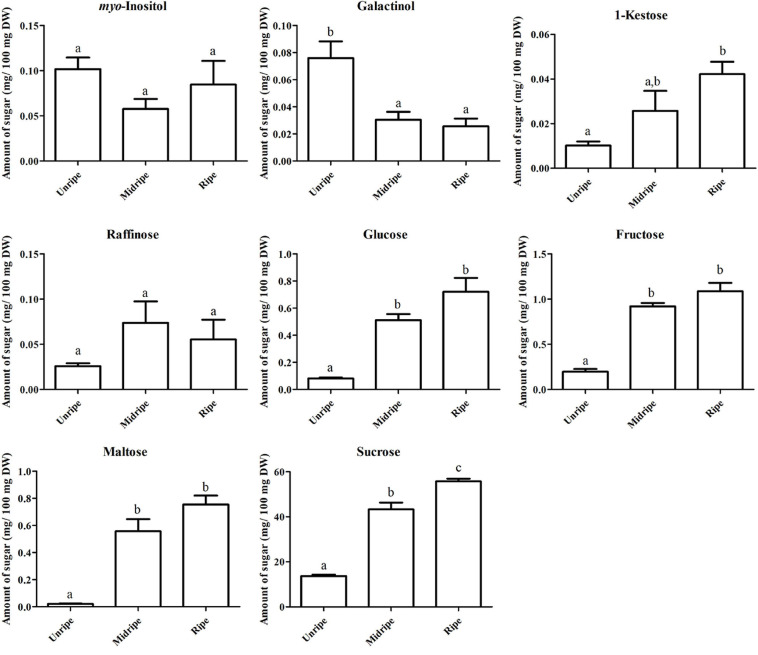
Soluble sugar content based on dry weight, including myo-inositol, galactinol, 1-kestose, raffinose, glucose, fructose, maltose, and sucrose contents (mg/100 mg dry weight [DW]) of the durian pulp at three ripening stages (including unripe, midripe, and ripe). Each bar indicates mean ± standard error (±SEM) values from triplicate experiments and significant difference between stages is denoted by lower case letters.

Sugar is an important metabolite group responsible for sweetness in fruits. Previously, sucrose, fructose, glucose, and maltose were increased in ripe durian pulp samples (Charoenkiatkul et al., 2018). Using GC-TOF/MS, we confirmed that these four major sugars and a newly annotated sugar, raffinose, were significantly increased during ripening with the log_2_ FC ranging between 1.16 and 4.84 (Figure 2), while the newly annotated 1-kestose was unchanged. Since sugars were only annotated from GC-TOF/MS in this work, we applied HPAEC-PAD analysis to validate and confirm the result (Figure 3). According to the results, the contents of all four major sugars agreed with GC-TOF/MS. However, the content of 1-kestose was significantly increased, while the content of raffinose remained unchanged during the ripening period (Figure 3). Disagreements between the two methods could be obtained when the concentrations in the samples were very low, as raffinose and 1-kestose were in this case. When using GC-MS, it is suggested that optimum derivatization and separation are needed before measuring sugars in each individual plant extract (Füzfai et al., 2004). Therefore, in our analysis, quantification by targeted HPAEC-PAD analysis is more reliable than the untargeted analysis, which can lead to the discovery of novel metabolites. Fruits can accumulate different types of sugars, but sucrose is the primary storage sugar in many fruits, such as melon, banana, peach, and strawberry (Yamaki, 2010). Notably, durian pulp contains over 50% sucrose by dry weight (Figure 3), which makes it the highest sucrose-accumulating fruit among those reported. Since sucrose is the most abundant sugar, this suggests that the major sweetness-providing metabolite in the ripe durian pulp is sucrose.

In addition, we identified two ripening-associated sugar alcohols found at low concentration related to raffinose, galactinol and *myo*-inositol (Figure 2). Galactinol content decreased during ripening, which was in agreement with the two analytical methods (Figure 3), whereas *myo*-inositol content remained unchanged, as confirmed by HPAEC-PAD. Raffinose and *myo*-inositol are synthesized from galactinol. The decrease in galactinol during the ripening period suggested that it might be stored before ripening then is converted to the downstream sugars later. The accumulation of raffinose-family sugars varies between various fruits (Jovanovic-Malinovska et al., 2014), and their role in fruits has not been studied much to date; however, they may be involved in stress tolerance during ripening. Furthermore, we found an increase in sugar phosphates, including glucosamine-6-phosphate, *N*-acetyl-*D*-galactosamine-6-phosphate, lactose-1-phosphate, sucrose-6-phosphate, and trehalose-6-phosphate in ripe durian pulp with log_2_ FC ranging between 1.34 and 2.09 (Figure 2). Sugar phosphates are intermediates in different biological pathways. In addition, they also act as signaling molecules. One interesting metabolite is trehalose-6-phosphate, which is an intermediate for trehalose biosynthesis. It is an important signaling molecule for sucrose consumption and a negative feedback molecule for sucrose biosynthesis in plants (Fichtner et al., 2020). Therefore, the increase in trehalose-6-phosphate in durian pulp possibly helps to control the sucrose content and supports trehalose content at the same time.

We detected 22 ripening-related organic acids in the durian pulp. Of these, the levels of 20 organic acids significantly increased during durian ripening, with log_2_ FC ranging between 1.38 and 10.33, while those of 2-isopropylmalate and threonate decreased (Log_2_ FC = −1.25 and −2.56, respectively) (Figure 2). There were five ripening-related organic acids which participated in central carbon pathways, namely *cis*-aconitate, fumarate, malate, pyruvate, and succinate. Malate and succinate were the highest (Supplementary Table 2), which was consistent with the results for the ripe durian pulps from other cultivars (Yi et al., 2020). Another abundant organic acid was citrate, and its content was not significantly changed during the ripening period (Supplementary Table 2). Therefore, malate and succinate probably contribute to sour taste in the ripe fruit, even if the sweet taste is dominant in the pulp, due to high sugar composition. In addition, we also detected a significant change in 4-methyl-2-oxovalerate and 2-isopropylmalate (Figure 2), which are two important intermediates in branched-chain amino acid metabolism that will be elaborated in the section below.

Amino acids serve as important flavor precursors in many fruits, including durian. We detected 31 ripening-associated amino acids, while three amino acids were unchanged: asparagine, glutamate, and threonine. Levels of 15 proteinogenic and 14 non-proteinogenic amino acids increased during durian ripening, with log_2_ FC ranging between 1.34–5.65 and between 1.4–9.15, respectively, while only aspartate and ornithine decreased (Log_2_ FC = −1.36 and −5.64, respectively) (Figure 2). Durian pulp contains several volatile sulfur compounds. The volatiles are associated with amino acids in sulfur-related pathways. Notably, cysteine is the most modulated amino acid in the pathway (Figure 2 and Supplementary Table 4) and is an important precursor for the biosynthesis of methionine, another sulfur-containing amino acid. We found that methionine levels slightly increased in ripening durian pulp (Figure 2 and Supplementary Table 4). Methionine is a central metabolite of the sulfur pathway and ethylene biosynthesis, in which ethylene is an important compound for climacteric ripening. Therefore, this may be the reason for the slight changes in methionine content during ripening. Methionine is also an important precursor for sulfur volatile production, providing strong odor. Previous research on melon demonstrated that exogenous apply of methionine increased the content of sulfur volatiles related to methanethiol (Gonda et al., 2013). Interestingly, methanethiol and other thiol volatiles can be detected at high concentrations in ripe durian pulp using static headspace gas chromatography-olfactometry (SHGC-O) (Li et al., 2012) and headspace solid-phase microextraction GC-MS (Teh et al., 2017). In addition, we also found the accumulation of serine and homoserine (Figure 2), which are intermediates in the sulfur pathway, supporting the involvement of the pathway during the ripening process. Therefore, the accumulation of these amino acids may provide key precursors that contribute to the characteristic sulfuryl odor of durian.

In addition to sulfuryl odor, durian pulp also exhibits a fruity aroma during ripening. Branched-chain amino acids, including isoleucine, leucine, and valine, are precursors for volatile alcohols, esters, aldehydes, and lipid-derived compounds (Roze et al., 2010). We found a significant increase in the levels of these three amino acids and the isoleucine-derived keto acid, 4-methyl-2-oxovalerate in ripe durian pulp (Figure 2). According to a previous study conducted on strawberry, exogenously applied isoleucine increased the level of ethyl 2-methylbutanoate, a volatile ester (Perez et al., 2002). Furthermore, either exogenously applied isoleucine or 4-methyl-2-oxovalerate elevated the levels of ethyl 2-methylbutanoate, while applying valine increased the ethyl 2-methylpropanoate level (Gonda et al., 2010). These two volatiles, providing fruity odor, were also identified at high concentrations in the durian pulp (Li et al., 2012). Therefore, the accumulation of branched-chain amino acids can be important for the biosynthesis of volatile esters, and this accumulation may affect the expression of durian’s fruity aroma during ripening.

For the aromatic amino acid pathway, phenylalanine, tryptophan, and tyrosine are ripening-related amino acids in durian fruit (Figure 2). These amino acids are precursors for secondary metabolites such as flavonoids, alkaloids, and indoles. Interestingly, durian pulp contains a high amount of flavonoids at the ripe stage, which positively correlates with the high antioxidant activity of the pulp (Haruenkit et al., 2010). Accumulation of these amino acids may be important for durian pulp to synthesize secondary metabolites during ripening.

Aspartate was the only proteinogenic amino acid whose levels decreased during ripening (Figure 2). It can be directly used for the biosynthesis of glutamate and asparagine. Moreover, it is involved in the biosynthesis of lysine, methionine, isoleucine, leucine, and valine, whose contents increased during ripening, and threonine, whose content was unchanged. Therefore, the decrease in aspartate levels during ripening could result from its usage for biosynthesis of other amino acids. Glutamate is a highly abundant amino acid that provides the umami taste in durian pulp (Pinsorn et al., 2018). We found that the glutamate content remained constant during the ripening period (Supplementary Table 2). Glutamate can be converted to other metabolites, such as glutamine, arginine, proline, and GABA (Sorrequieta et al., 2010). We observed increased levels of proline and hydroxyproline during ripening. Until now, the roles of these two metabolites in fruits have not been well understood. Previous studies have shown that accumulation of proline is involved in the nitrogen sink mechanism, and it helps to increase the fruit quality traits and yield (Sayed et al., 2014). Regarding the precursors for polyamine biosynthesis, we found that the arginine content was unchanged (Supplementary Table 2), while ornithine content decreased during the ripening period (Figure 2). Both amino acids can be converted to putrescine, a central metabolite of the polyamine pathway.

For polyamines, we found significant increases in *N*^8^-acetylspermidine and putrescine, while spermidine and spermine were unchanged and decreased, respectively (Figure 2 and Supplementary Table 2). Polyamines are associated with abiotic stress responses in fruits (Chen et al., 2019). Furthermore, previous studies have shown that they are also involved in the modulation of fruit ripening by intricate crosstalk with the ethylene pathway (Tassoni et al., 2006). The role of polyamines in durian pulp still requires further investigation, and their accumulation might be related to the ripening period of durian. Polyamines are also precursors for biosynthesis of GABA, a health-promoting bioactive compound. Interestingly, GABA was highly enriched in ripe durian pulp (Supplementary Table 4 and Figure 2). Although the role of GABA accumulation in fruit has not been clearly ascertained, previous studies have shown that it is associated with abiotic stress responses in strawberry (Deewatthanawong et al., 2010), tomato (Wu et al., 2020), and apple (Brikis et al., 2018). It has been shown that these fruits accumulate high GABA content under elevated CO_2_ and low O_2_. Therefore, the role of GABA in durian might also be involved in stress response. Further studies in durian may help to promote its market value by developing appropriate storage conditions for higher GABA content in the future.

The levels of nine nucleotide derivatives increased during ripening, with log_2_ FC ranging between 1.32 and 8.28 (Figure 2), while those of cytidine diphosphate (CDP) and deoxyguanosine decreased (Log_2_ FC = −2.06 and −2.18, respectively). To our knowledge, 3 of 11 nucleotide derivatives are known to be involved in fruit flavor. AMP, IMP, and GMP accumulation is associated with enhancing the umami taste in tomato (Chew et al., 2017). In addition, we also detected 14 additional miscellaneous metabolites with significantly altered levels during durian ripening with log_2_ FC ranging between −2.18 and 8.45 (Figure 2). Some of these metabolites are food supplements in the durian pulp, such as gluconate and pyridoxamine, the so-called vitamin B group (Pinsorn et al., 2018).

### Transcriptome Analysis of Durian cv. Monthong During Ripening

The metabolomic data provided us only the ripening-associated metabolites and the pathways associated with durian flavor. Therefore, to understand the biochemical modification of the pathways at a deeper and more comprehensive level, we integrated transcriptome analysis to study the expression of candidate genes in associated pathways.

Approximately, 10–16 million clean reads from unripe and ripe Monthong were generated. The clean reads were then used to perform *de novo* assembly, which generated 164,618 transcripts with an average length of 1,082.68 bp, and a total of 85,164 coding regions were predicted. Afterward, total reads of each library were mapped to the *de novo* assembled transcriptome (Supplementary Table 5). The annotation results of transcripts are shown in Supplementary Table 6. The number of the transcripts obtained in this study is similar to the previous report by Teh et al. (2017). The PCA plot shows that the expression of transcripts in the unripe samples were clearly separated from the ripe samples according to PC1 (90.1%) (Supplementary Figure 5), suggesting that gene expression changes as the ripening progresses. DEG analysis identified a total of 18,616 DEGs (Supplementary Table 7) with 8,145 upregulated genes and 10,471 downregulated genes. Subsequently, we identified the top 10 upregulated genes by FC. Two of those genes were polygalacturonase (PG) and 1-aminocyclopropane-1-carboxylate synthase (ACS) (Supplementary Table 7), which are responsible for cell wall degradation and ethylene biosynthesis, respectively. In addition, we identified a key sulfur metabolism gene, methionine gamma-lyase (MGL), which contributes to VSC biosynthesis in sulfur metabolism (Teh et al., 2017). Although MGL did not have a high FC value, it showed a remarkably high RPKM expression value (Supplementary Table 8), suggesting its importance during durian ripening. Additionally, a high RPKM value was also found for 1-aminocyclopropane-1-carboxylate oxidase (Supplementary Table 8) at the ripe stage. DEGs were then used to perform GO analysis, and most of the GO terms were assigned to biological processes. Within biological processes, the largest proportion was in protein modification by small protein conjugation and ubiquitin-dependent protein catabolic process categories, respectively. Within molecular function, the largest proportion was in the ATPase activity category. Lastly, within the cellular compartment, the largest proportion was in the host cell nucleus category (Supplementary Figure 6). To reveal the biological pathways associated with DEG data, DEGs were assigned to KEGG. The results showed that the DEGs participated in 144 biological pathways. The top five pathways are purine metabolism, thiamine metabolism, starch and sucrose metabolism, glycolysis/gluconeogenesis, and pyruvate metabolism (Supplementary Figure 7). This result is consistent with the metabolic pathway analysis which found that the change of nucleotide metabolism, sugar metabolism, and central carbon metabolism was highly associated with the durian ripening process.

**FIGURE 4:**
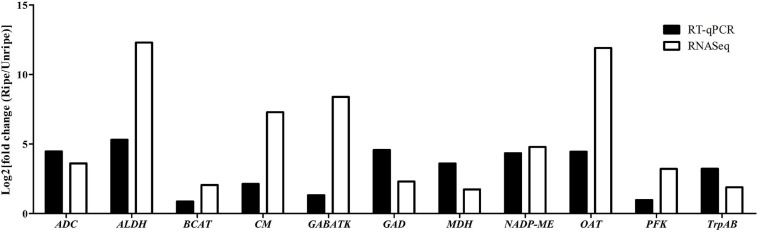
Log_2_ fold change in expression values in the ripe pulps relative to unripe pulps, as measured with RNA-seq and RT-qPCR in the 11 selected genes.

To validate the DEGs identified from RNA-seq results, we randomly selected 11 key genes (*ALDH* [LOC111284558], *ADC* [LOC111308806], *CM* [LOC111286253], *GABA-TK* [LOC111305652], *GAD* [LOC111281776], *BCAT* [LOC111305652], *MDH* [LOC111293205], *NADP-MEs* [LOC111286064], *OAT* [LOC111278083], *PFK* [LOC111285266], and *TrpAB* [LOC111287933]) from various metabolic pathways possibly related to durian flavor, and their expression levels were analyzed by RT-qPCR using Monthong cDNA at the unripe and ripe stages. According to the RT-qPCR analysis, the expression results of all selected genes agreed with results obtained from RNA-seq analysis showing the reliability of the RNA-seq method for expression quantitation (Figure 4).

### Identification of Candidate Genes in Metabolic Pathways Contributing to Durian Flavor

**FIGURE 5:**
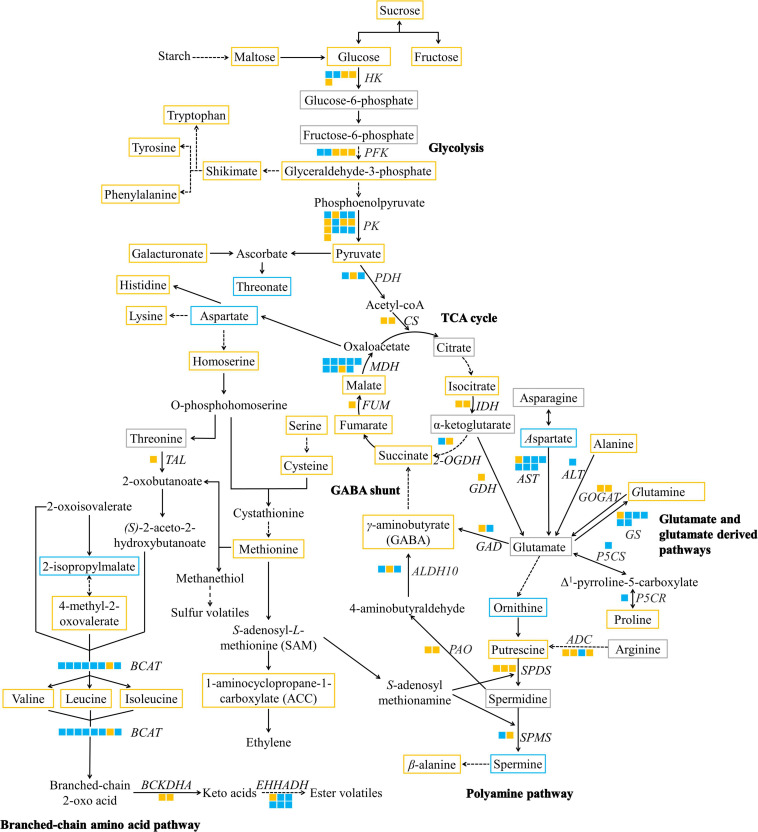
A metabolic map of significantly altered metabolites and DEGs of taste-associated pathways during durian cv. Monthong ripening. Metabolites with significantly increased, significantly decreased, and unchanged contents are outlined in orange, blue, and gray, respectively. Undetectable metabolites are shown without boxes. Isoforms of each putative gene are represented by a solid box, and each isoform is arranged by locus number. Upregulated and downregulated isoforms are colored in orange and blue, respectively. Solid arrows indicate one enzymatic reaction, while dash arrows indicate multiple reactions. *HK*, hexokinase; *PFK*, phosphofructokinase; *PK*, pyruvate kinase*; PDH*, pyruvate dehydrogenase; *CS*, citrate synthase; *IDH*, isocitrate dehydrogenase; *2-OGDH*, α-ketoglutarate dehydrogenase; *FUM*, fumarase; *MDH*, malate dehydrogenase; *AST*, aspartate aminotransferase; *ALT*, alanine aminotransferase; *GDH*, glutamate dehydrogenase; *GOGAT*, glutamate synthase; *GS*, glutamine synthetase; *P5CS, Δ^1^-pyrroline-5-carboxylate synthetase*; *P5CR*, Δ^1^-pyrroline-5-carboxylate reductase; *ADC*, arginine decarboxylase; *SPDS*, spermidine synthase; *SPMS*, spermine synthase; *GAD*, glutamate decarboxylase; *PAO*, polyamine oxidase; *ALDH10*, 4-aminobutanal dehydrogenase; *TAL*, threonine ammonia-lyase; *BCAT, branched-chain amino acid transaminase*; *BCKDHA*, 3-methyl-2-oxobutanoate dehydrogenase; *EHHADH*, long-chain-enoyl-CoA hydratase.

Our results demonstrated that sugar and central carbon pathways (glycolysis and TCA cycle) are important pathways during the ripening process (Supplementary Figure 7). High sugar accumulation promotes sweet taste in ripe durian pulp. Alternatively, sugars are metabolized into the central carbon pathway, which not only produces cellular energy, but also generates organic acids for fruit acidic taste and precursors for amino acid biosynthesis. In this section, we focused on the modulation of the central carbon pathway and its connection to flavor-related pathways. Therefore, a metabolic network was constructed to elucidate the relationship between changes in metabolite levels and candidate genes (Figure 5). Information related to candidate genes, such as locus number and expression level, are provided in Supplementary Table 8.

### Central Carbon Pathway

We identified genes in the glycolytic pathway, including hexokinase (*HK*), phosphofructokinase (*PFK*), and pyruvate kinase (*PK*), which control rate-limiting steps of the glycolytic pathway in fruit (Ball et al., 1991). According to our results, some isoforms, annotated as *HK*, *PFK*, and *PK*, were upregulated with high RPKM values at the ripe stage compared to the downregulated isoforms (Figure 5 and Supplementary Table 8). The expression of these isoforms is consistent with the accumulation of pyruvate, the final product of the pathway (Figure 5), suggesting that these candidate genes participate in glycolysis during durian ripening. Afterward, pyruvate is converted to acetyl-CoA and enters the TCA cycle by pyruvate dehydrogenase (*PDH*), whose isoform was annotated as *PDH* acyl-transferring subunit was upregulated in the ripe durian pulp (Figure 5 and Supplementary Table 8). The first step of the TCA cycle is producing citrate, which is controlled by citrate synthase (*CS*). Although *CS* was upregulated, the content of citrate remained constant during ripening (Figure 5), suggesting that the rate of citrate biosynthesis is probably equal to citrate consumption in the durian. Furthermore, we also observed the upregulation of other genes in the TCA cycle, including isocitrate dehydrogenase (*IDH*), α-ketoglutarate dehydrogenase (*2-OGDH*), fumarase (*FUM*), and malate dehydrogenase (*MDH*), together with the upregulated metabolites, isocitrate, succinate, fumarate, and malate (Figure 5). These results show the activation of the TCA cycle, supporting citrate biosynthesis during ripening. Citrate is used in glutamate metabolism and is bypassed through α-ketoglutarate. We found that the content of α-ketoglutarate was unchanged during the ripening period. Notably, the gene for its biosynthesis, *IDH*, the gene for its degradation, *2-OGDH*, and glutamate dehydrogenase (*GDH*), were upregulated (Figure 5). This result suggests that α-ketoglutarate content remains constant because it is converted for the TCA cycle by *2-OGDH* and bypassed to glutamate metabolism by *GDH*. Similar observations were found in other fruits, such as tomato (Yin et al., 2010), showing that the change in citrate is correlated to α-ketoglutarate content during ripening and salt-stress treatment. Although some metabolites in this pathway do not provide the dominant taste found in ripe durian, they serve as important precursors for the biosynthesis of other taste-related compounds, which will be discussed later.

### Glutamate and Glutamate-Derived Metabolic Pathways

Glutamate, an amino acid providing umami taste, is linked to the TCA cycle via α-ketoglutarate, which is controlled by *GDH*. We found that the gene was upregulated during the ripening period (Figure 5 and Supplementary Table 8). Additionally, there are three routes for glutamate biosynthesis, including aspartate aminotransferase (*AST*), alanine aminotransferase (*ALT*), and glutamate synthase (*GOGAT*). We found that only *AST* and *GOGAT* were upregulated during the ripening period (Figure 5). Notably, the route from *AST* (LOC111284590) contained the highest log_2_ FC (4.83) and RPKM expression value compared to the other routes (Supplementary Table 8). Therefore, this route is highly activated for glutamate biosynthesis during durian ripening. Glutamate interconverts with aspartate, and this step is controlled by *AST*. We found that the decrease in aspartate content during durian ripening correlated with the upregulation of *AST* (Figure 5), suggesting a transfer of the amino group from aspartate to glutamate.

Although the above-mentioned glutamate-related biosynthetic genes were highly upregulated, glutamate content remained unchanged during the ripening period (Figure 5), implying an equal rate of biosynthesis and consumption. Interestingly, we found upregulation of several genes related to glutamate conversion. *GS* was correlated with an increase in glutamine content (Figure 5). Furthermore, we also found the upregulation of nitrate reductase (*NR*) (Supplementary Table 8). It has been reported that GS and NR were upregulated under higher nitrogen supply, resulting in higher accumulation of soluble sugars, free amino acids, and other substances related to fruit quality (Scarpeci et al., 2007; Liao et al., 2019). Therefore, activating the nitrogen metabolism in ripening durian fruits could be important for enhancing taste-related characteristics. Glutamate can also be sequentially converted to Δ^1^-pyrroline-5-carboxylate and proline by a dual activity enzyme, possessing of Δ^1^-pyrroline-5-carboxylate synthetase (*P5CS*) and Δ^1^-pyrroline-5-carboxylate reductase (*P5CR*). Only one isoform of the gene was found in our transcriptomic data, and it was downregulated during durian ripening, which was not consistent with the increased proline content (Figure 5). A previous report showed that proline content may not be correlated to the expression of *P5CR* in developing tissues of Arabidopsis (Hua et al., 1997). Therefore, this isoform may be the only candidate genes of proline metabolism in the pulp. In addition, we also identified proline dehydrogenase, a gene involved in proline catabolism, was downregulated during durian ripening (Supplementary Table 8).

Furthermore, glutamate can be used for the biosynthesis of ornithine and arginine, which are precursors for polyamine biosynthesis. These two amino acids can later be converted to putrescine, a precursor for further polyamine production. We propose that the primary route for polyamine biosynthesis in durian pulp is through arginine route because the key gene arginine decarboxylase (*ADC*) was upregulated with high RPKM value during durian ripening (Figure 5 and Supplementary Table 8), while the expression level of ornithine decarboxylase (*ODC*), controlling the reaction from ornithine to putrescine, was not significantly changed. The arginine route has been found to be a major pathway for polyamine biosynthesis in other fruits such as apple and tomato (Hao et al., 2005). For the polyamine pathway, spermidine synthase (*SPDS*) and spermine synthase (*SPMS*) control steps that convert putrescine to spermidine and spermine, respectively. We found upregulation of *SPDS* and *SPMS* during durian ripening (Figure 5), which were not correlated with spermidine and spermine content. Spermidine is either converted to 4-aminobutyraldehyde or is used for spermine biosynthesis. Moreover, spermine can be degraded by β-alanine metabolism. Therefore, these are probably the reasons for the unchanged spermidine content and decreased spermine content during the ripening period.

GABA is a bioactive compound with a biosynthetic pathway that bypasses from α-ketoglutarate. Interestingly, we found a significant increase in GABA levels in the ripe durian pulp (Figure 2). There are two main GABA biosynthetic pathways in fruits, which are GABA shunts and alternative pathways from polyamines. For the GABA shunt, we found the upregulation of glutamate decarboxylase (*GAD*) in ripening durian pulp, which is positively correlated with GABA content (Figure 5). GABA shunts are the major pathway in many fruits. *GAD* is the key gene that controls the conversion from glutamate to GABA in this pathway. Similar to results from citrus fruit, the activation of GABA shunt is also related to increased transcription of *GDH*, *AST*, and *GS*, which is a major route for citrate catabolism (Katz et al., 2011; Sadka et al., 2019). These findings are consistent with our results in ripening durian. In addition, we found the upregulation of several genes in polyamine-derived-GABA biosynthesis, including polyamine oxidase (*PAO*) and 4-aminobutanal dehydrogenase (*ALDH10*). Therefore, our gene expression results supported the theory that two pathways contribute to GABA production during durian ripening.

### Branched-Chain Amino Acid Pathway

An overpowering smell is a well-known characteristic of ripe durian. Besides the sulfuryl aroma, ripe durian also contains a sweet, fruity aroma, and the volatiles providing such aroma are associated with branched-chain amino acid metabolism. Interestingly, we observed high upregulation of threonine ammonia-lyase (*TAL*) (Log_2_ FC = 5.52) (Supplementary Table 8), which converts threonine to 2-oxobutanoate, an important intermediate for BCAA biosynthesis, during durian ripening (Figure 5). Moreover, we also observed high upregulation of genes in the BCAA pathway, such as branched-chain amino acid aminotransferase (*BCAT*) (Log_2_ FC = 6.32) (Supplementary Table 8), which is a key regulatory gene controlling branched-chain amino acid biosynthesis and degradation (Gonda et al., 2010; Maloney et al., 2010), supporting the activation of this pathway. In addition, branched-chain amino acids are precursors for volatile ester production. Notably, the upregulation of 3-methyl-2-oxobutanoate dehydrogenase (*BCKDHA*) and long-chain-enoyl-CoA hydratase (*EHHADH*), which are key genes in pathways connected to branched-chain amino acid degradation (Kochevenko et al., 2012), was observed during durian ripening (Figure 5 and Supplementary Table 8). Our results support the hypothesis that branched-chain amino acid biosynthesis is upregulated during durian ripening, and that these amino acids are possibly used for volatile ester production, providing the aroma of ripe durian.

In conclusion, our findings provide fundamental knowledge for future durian molecular studies. We expect that these findings will allow comparison of the candidate gene expression and their related metabolites among different durian cultivars, thereby assisting in developing molecular markers for durian breeding that may prove useful the future.

## Data Availability Statement

The original contributions presented in the study are publicly available. This data can be found here: The raw sequences have been deposited into NCBI Sequence Read Archive (SRA) under the project accession number PRJNA683229 and CNGB Sequence Archive (CNSA) of China National GeneBank DataBase (CNGBdb) with project accession number CNP0001432.

## Author Contributions

SS conceived the original screening and research plan. LS wrote the first draft of the manuscript. AO and RS performed CE-TOF/MS analysis. AE and JK performed GC-TOF/MS analysis. LS and KW performed HPAEC-PAD analysis. LS performed *de novo* transcriptome analysis. LS and PP analyzed the data. LS, GK, PP, AO, AE, JK, RH, KW, MW, TT, KS, and SS revised the manuscript. All authors read and approved the final manuscript.

## Conflict of Interest

The authors declare that the research was conducted in the absence of any commercial or financial relationships that could be construed as a potential conflict of interest.

## Footnotes

1 https://www.metaboanalyst.ca/

## References

[B1] Aziz N. A. A. Jalil A. M. M. (2019). Bioactive compounds, nutritional value, and potential health benefits of indigenous durian (Durio zibethinus Murr.): A review. *Foods.* 8 96. 10.3390/foods8030096 30871187 PMC6463093

[B2] Ball K. L. Green J. H. Rees T. (1991). Glycolysis at the climacteric of bananas. *Eur. J. Biochem.* 197 265–269. 10.1111/j.1432-1033.1991.tb15907.x 1849821

[B3] Brikis C. J. Zarei A. Chiu G. Z. Deyman K. L. Liu J. Trobacher C. P. (2018). Targeted quantitative profiling of metabolites and gene transcripts associated with 4-aminobutyrate (GABA) in apple fruit stored under multiple abiotic stresses. *Hortic. Res.* 5 61. 10.1038/s41438-018-0069-3 30510768 PMC6269452

[B4] Carrari F. Fernie A. R. (2006). Metabolic regulation underlying tomato fruit development. *J. Exp. Bot.* 57 1883–1897. 10.1093/jxb/erj020 16449380

[B5] Charoenkiatkul S. Thiyajai P. Judprasong K. (2018). Nutrients and bioactive compounds in popular and indigenous durian (Durio zibethinus Murr.). *Food Chem.* 193 181–186. 10.1016/j.foodchem.2015.02.107 26433306

[B6] Chen D. Shao Q. Yin L. Younis A. Zheng B. (2019). Polyamine function in plants: Metabolism, regulation on development, and roles in abiotic stress responses. *Front. Plant Sci.* 9:1945. 10.3389/fpls.2018.01945 30687350 PMC6335389

[B7] Chew B. L. Fisk I. D. Fray R. Tucker G. A. Bodi Z. Ferguson A. (2017). The effect of adenosine monophosphate deaminase overexpression on the accumulation of umami-related metabolites in tomatoes. *Plant Cell Rep.* 36 81–87. 10.1007/s00299-016-2058-z 27662835

[B8] Deewatthanawong R. Nock J. F. Watkins C. B. (2010). γ-Aminobutyric acid (GABA) accumulation in four strawberry cultivars in response to elevated CO2 storage. *Postharvest Biol. Technol.* 57 92–96. 10.1016/j.postharvbio.2010.03.003

[B9] Erban A. Martinez-Seidel F. Rajarathinam Y. Dethloff F. Orf I. Fehrle I. (2020). “Multiplexed Profiling and Data Processing Methods to Identify Temperature-Regulated Primary Metabolites Using Gas Chromatography Coupled to Mass Spectrometry,” in *Plant Cold Acclimation: Methods in Molecular Biology (Methods and Protocols)*, eds Hincha D. K. Zuther E. (Louisville, KY: Humana), 203–239. 10.1007/978-1-0716-0660-5_15 32607984

[B10] Fichtner F. Barbier F. F. Annunziata M. G. Feil R. Olas J. J. Mueller-Roeber B. (2020). The role of trehalose 6-phosphate in shoot branching – local and non-local effects on axillary bud outgrowth in Arabidopsis rosettes. *New Phytol.* 229 2135–2151. 10.1111/nph.17006 33068448

[B11] Füzfai Z. Katona Z. F. Kovács E. Molnár-Perl I. (2004). Simultaneous identification and quantification of the sugar, sugar alcohol, and carboxylic acid contents of sour cherry, apple, and berry fruits, as their trimethylsilyl derivatives, by gas chromatography-mass spectrometry. *J. Agric. Food Chem.* 52 7444–7452. 10.1021/jf040118p 15675786

[B12] Gao J. Zhang Y. Li Z. Liu M. (2020). Role of ethylene response factors (ERFs) in fruit ripening. *J. Food. Qual.* 4 15–20. 10.1093/fqsafe/fyz042

[B13] Gonda I. Bar E. Portnoy V. Lev S. Burger J. Schaffer A. A. (2010). Branched-chain and aromatic amino acid catabolism into aroma volatiles in Cucumis melo L. fruit. *J. Exp. Bot.* 61 1111–1123. 10.1093/jxb/erp390 20065117 PMC2826658

[B14] Gonda I. Lev S. Bar E. Sikron N. Portnoy V. Davidovich-Rikanati R. (2013). Catabolism of L-methionine in the formation of sulfur and other volatiles in melon (Cucumis melo L.) fruit. *Plant J.* 74 458–472. 10.1111/tpj.12149 23402686

[B15] Guo Y. S. Tao J. Z. (2018). Metabolomics and pathway analyses to characterize metabolic alterations in pregnant dairy cows on D 17 and D 45 after AI. *Sci. Rep.* 8 5973. 10.1038/s41598-018-23983-2 29654235 PMC5899158

[B16] Hao Y. Kitashiba H. Honda C. Nada K. Moriguchi T. (2005). Expression of arginine decarboxylase and ornithine decarboxylase genes in apple cells and stressed shoots. *J.Exp. Bot* 56 1105–1115. 10.1093/jxb/eri102 15723827

[B17] Haruenkit R. Poovarodom S. Vearaslip S. Namiesnik J. Sliwka-Kaszynska M. Park Y. (2010). Comparison of bioactive compounds, antioxidant and antiproliferative activities of Mon Thong durian during ripening. *Food Chem.* 118 540–547. 10.1016/j.foodchem.2009.05.029

[B18] Hua X. J. Cotte B. V. Montagu M. V. Verbruggen N. (1997). Developmental regulation of pyrroline-5-carboxylate reductase gene expression in Arabidopsis. *Plant Physiol.* 114 1215–1224. 10.1104/pp.114.4.1215 9276946 PMC158414

[B19] Jovanovic-Malinovska R. Kuzmanova S. Winkelhausen E. (2014). Oligosaccharide profile in fruits and vegetables as sources of prebiotics and functional foods. *Int. J. Food Prop.* 17 949–965. 10.1080/10942912.2012.680221

[B20] Katz E. Boo K. H. Kim H. Y. Eigenheer R. A. Phinney B. S. Shulaev V. (2011). Label-free shotgun proteomics and metabolite analysis reveal a significant metabolic shift during citrus fruit development. *J. Exp. Bot.* 62 5367–5384. 10.1093/jxb/err197 21841177 PMC3223037

[B21] Ketsa S. Daengkanit T. (1998). Physiological changes during postharvest ripeningof durian fruit (Durio zibethinus Murray). *J. Hortic. Sci. Biotechnol.* 73 575–577. 10.1080/14620316.1998.11511017

[B22] Khaksar G. Sangchay W. Pinsorn P. Sangpong L. Sirikantaramas S. (2019). Genome wide analysis of the Dof gene family in durian reveals fruit ripening-associated and cultivar-dependent Dof transcription factors. *Sci. Rep.* 9 12109. 10.1038/s41598-019-48601-7 31431665 PMC6702166

[B23] Khaksar G. Sirikantaramas S. (2020). Auxin response factor 2A is part of the regulatory network mediating fruit ripening through auxin-ethylene crosstalk in durian. *Front. Plant Sci.* 11:543747. 10.3389/fpls.2020.543747 33013965 PMC7509138

[B24] Kochevenko A. Araujo W. L. Maloney G. S. Tieman D. M. Do P. T. Taylor M. G. (2012). Catabolism of branched chain amino acids supports respiration but not volatile synthesis in tomato fruits. *Mol. Plant.* 5 366–375. 10.1093/mp/ssr108 22199237

[B25] Kopka J. Schauer N. Krueger S. Birkemeyer C. Usadel B. Bergmuller E. (2005). GMD@CSB.DB: the Golm Metabolome Database. *Bioinformatics.* 21 1635–1638. 10.1093/bioinformatics/bti236 15613389

[B26] Li J. Schieberle P. Steinhaus M. (2012). Characterization of the major odor-active compounds in Thai durian (Durio zibethinus L. ‘Monthong’) by aroma extract dilution analysis and headspace gas chromatography–olfactometry. *J. Agric. Food Chem.* 60 11253–11262. 10.1021/jf303881k 23088286

[B27] Liao L. Dong T. Liu X. Dong Z. Qiu X. Rong Y. (2019). Effect of nitrogen supply on nitrogen metabolism in the citrus cultivar ‘Huangguogan’. *PLoS ONE.* 14:e0213874. 10.1371/journal.pone.0213874 30897177 PMC6428318

[B28] Livak K. J. Schmittgen T. D. (2001). Analysis of relative gene expression data using real time quantitative PCR and the 2–ΔΔCt method. *Methods.* 25 402–408. 10.1006/meth.2001.1262 11846609

[B29] Lombardo V. A. Osorio S. Borsani J. Lauxmann M. A. Bustamante C. A. Budde C. O. (2011). Metabolic profiling during peach fruit development and ripening reveals the metabolic networks that underpin each developmental stage. *Plant Physiol.* 157 1696–1710. 10.1104/pp.111.186064 22021422 PMC3327199

[B30] Luedemann A. Strassburg K. Erban A. Kopka J. (2008). TagFinder for the quantitative analysis of gas chromatography - mass spectrometry (GC-MS) based metabolite profiling experiments. *Bioinformatics.* 24 732–737. 10.1093/bioinformatics/btn023 18204057

[B31] Maloney G. S. Kochevenko A. Tieman D. M. Tohge T. Krieger U. Zamir D. (2010). Characterization of the branched-chain amino acid aminotransferase enzyme family in tomato. *Plant Physiol.* 153 925–936. 10.1104/pp.110.154922 20435740 PMC2899903

[B32] Oikawa A. Fujita N. Horie R. Saito K. Tawaraya K. (2011). Solid-phase extraction for metabolomic analysis of high-salinity samples by capillary electrophoresis–mass spectrometry. *J. Sep. Sci.* 34 1063–1068. 10.1002/jssc.201000890 21416606

[B33] Pang Z. Zhou G. Chong J. Xia J. (2021). Comprehensive Meta-Analysis of COVID-19 Global Metabolomics Datasets. *Metabolites.* 11 44. 10.3390/metabo11010044 PMC7827862 33435351

[B34] Perez A. G. Olias R. Luaces P. Sanz C. (2002). Biosynthesis of strawberry aroma compounds through amino acid metabolism. *J. Agric. Food Chem.* 50 4037–4042. 10.1021/jf011465r 12083879

[B35] Pinsorn P. Oikawa A. Watanabe M. Sasaki R. Ngamchuachit P. Hoefgen R. (2018). Metabolic variation in the pulps of two durian cultivars: Unraveling the metabolites that contribute to the flavor. *Food Chem.* 268 118–125. 10.1016/j.foodchem.2018.06.066 30064738

[B36] Roze L. V. Chanda A. Laivenieks M. Beaudry R. M. Artymovich K. A. Koptina A. V. (2010). Volatile profiling reveals intracellular metabolic changes in Aspergillus parasiticus: VeA regulates branched chain amino acid and ethanol metabolism. *BMC Biochem.* 11:33. 10.1186/1471-2091-11-33 20735852 PMC2939540

[B37] Sadka A. Shlizerman L. Kamara I. Blumwald E. (2019). Primary metabolism in citrus fruit as affected by its unique structure. *Front. Plant Sci.* 10:1167. 10.3389/fpls.2019.01167 31611894 PMC6775482

[B38] Sadler G. D. Murphy P. A. (2010). “pH and Titratable Acidity,” in *Food Analysis. Food Analysis*, ed. Nielsen S. S. (Cham: Springer), 219–238. 10.1007/978-1-4419-1478-1_13

[B39] Sayed O. M. Gammal O. E. Salama A. (2014). Effect of proline and tryptophan amino acids on yield and fruit quality of Manfalouty pomegranate variety. *Sci. Hortic.* 169 1–5. 10.1016/j.scienta.2014.01.023

[B40] Scarpeci T. E. Marro M. L. Bortolotti S. Boggio S. B. Valle E. M. (2007). Plant nutritional status modulates glutamine synthetase levels in ripe tomatoes (Solanum lycopersicum cv. Micro-Tom). *J. Plant Physiol.* 164 137–145. 10.1016/j.jplph.2006.01.003 16513209

[B41] Sorrequieta A. Ferraro G. Boggio S. B. Valle E. M. (2010). Free amino acid production during tomato fruit ripening: a focus on L-glutamate. *Amino Acids.* 38 1523–1532. 10.1007/s00726-009-0373-1 19876714

[B42] Tan P. F. Ng S. K. Tan T. B. Chong G. H. Tan C. P. (2019). Shelf life determination of durian (Durio zibethinus) paste and pulp upon high pressure processing. *Food Res.* 3 221–230. 10.26656/fr.2017.3(3).215

[B43] Tassoni A. Watkins C. B. Davies P. J. (2006). Inhibition of the ethylene response by 1-MCP in tomato suggests that polyamines are not involved in delaying ripening, but may moderate the rate of ripening or over-ripening. *J. Exp. Bot.* 57 3313–3325. 10.1093/jxb/erl092 16920766

[B44] Teh B. T. Lim K. Yong C. H. Ng C. C. Y. Rao S. R. Rajasegaran V. (2017). The draft genome of tropical fruit durian (Durio zibethinus). *Nat. Genet.* 49 1633–1641. 10.1038/ng.3972 28991254

[B45] Tieman D. M. Zeigler M. Schmelz E. A. Taylor M. G. Bliss P. Kirst M. (2006). Identification of loci affecting flavour volatile emissions in tomato fruits. *J. Exp. Bot*. 57 887–896. 10.1093/jxb/erj074 16473892

[B46] Wu X. Jia Q. Ji S. Gong B. Li J. Lu G. (2020). Gamma-aminobutyric acid (GABA) alleviates salt damage in tomato by modulating Na+ uptake, the GAD gene, amino acid synthesis and reactive oxygen species metabolism. *BMC Plant Biol.* 20:465. 10.1186/s12870-020-02669-w 33036565 PMC7547442

[B47] Yamaki S. (2010). Metabolism and accumulation of sugars translocated to fruit and their regulation. *J. Jpn. Soc. Hortic. Sci.* 79 1–15. 10.2503/jjshs1.79.1

[B48] Yi T. X. Misran A. Whye C. K. Daim L. D. J. Ding P. Dek M. S. P. (2020). Postharvest quality indices of different durian clones at ripening stage and their volatile organic compounds. *Sci. Hortic.* 264 109169. 10.1016/j.scienta.2019.109169

[B49] Yin Y. Tominaga T. Iijima Y. Aoki K. Shibata D. Ashihara H. (2010). Metabolic alterations in organic acids and γ-aminobutyric acid in developing tomato (Solanum lycopersicum L.) fruits. *Plant Cell Physiol.* 51 1300–1314. 10.1093/pcp/pcq090 20595461

[B50] Zhu G. Wang S. Huang Z. Zhang S. Liao Q. Zhang C. (2018). Rewiring of the fruit metabolome in tomato breeding. *Cell.* 172 249–261. 10.1016/j.cell.2017.12.019 29328914

