# Supplementary material for: Assessing Dynamic Changes of Taste-Related Primary Metabolism During Ripening of Durian Pulp Using Metabolomic and Transcriptomic Analyses
Source: Front Plant Sci. 2021 Jun 18;12:687799. doi: 10.3389/fpls.2021.687799 (PMC8250156; doi:10.3389/fpls.2021.687799)
Supplement: Supplementary file 2 [file Presentation_1.PPTX]

## Slide 1
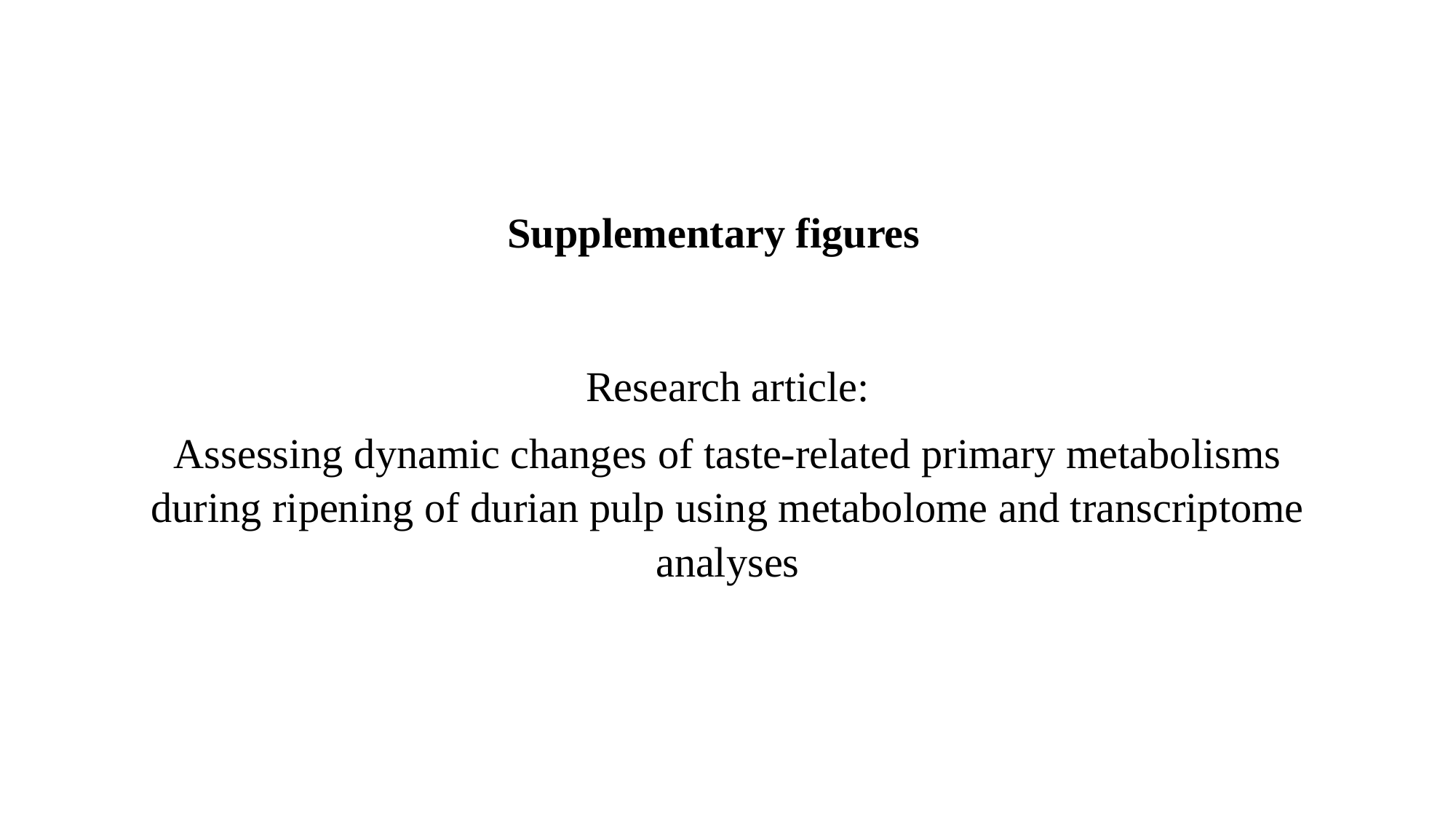

Supplementary figures
Research article:
Assessing dynamic changes of taste-related primary metabolisms during ripening of durian pulp using metabolome and transcriptome analyses

## Slide 2
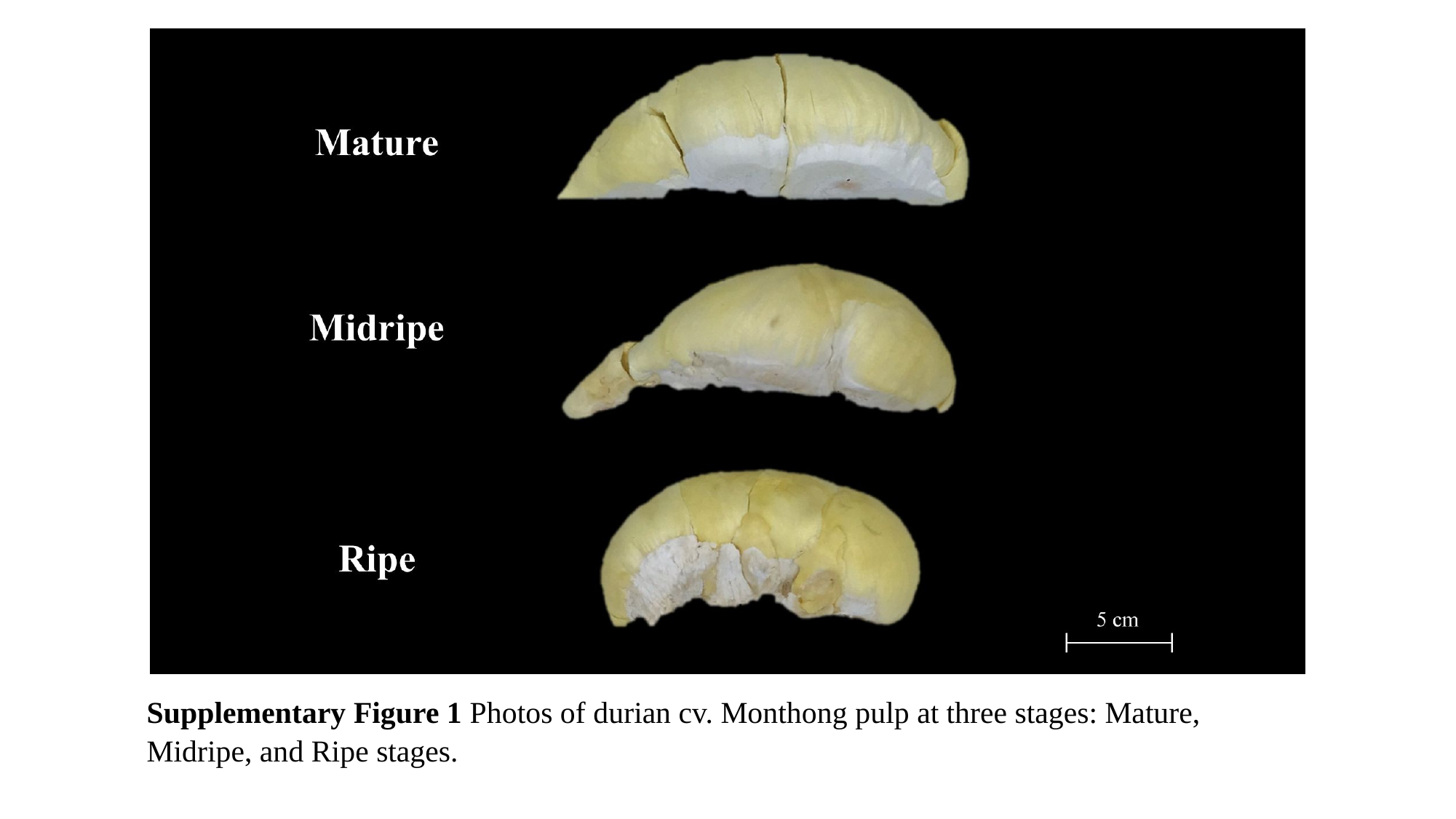

Supplementary Figure 1 Photos of durian cv. Monthong pulp at three stages: Mature, Midripe, and Ripe stages.

## Slide 3
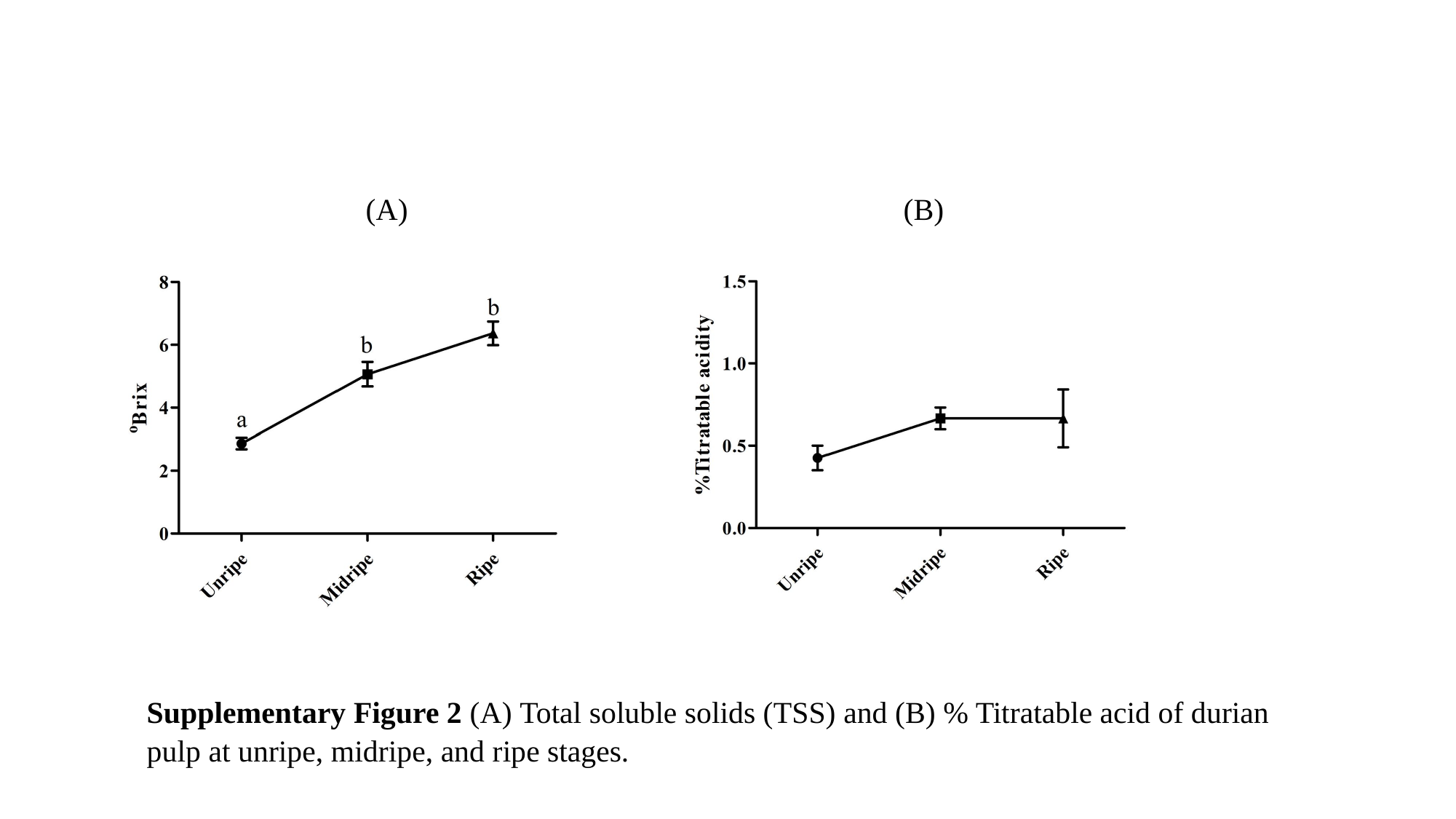

(A)
(B)
Supplementary Figure 2 (A) Total soluble solids (TSS) and (B) % Titratable acid of durian pulp at unripe, midripe, and ripe stages.

## Slide 4
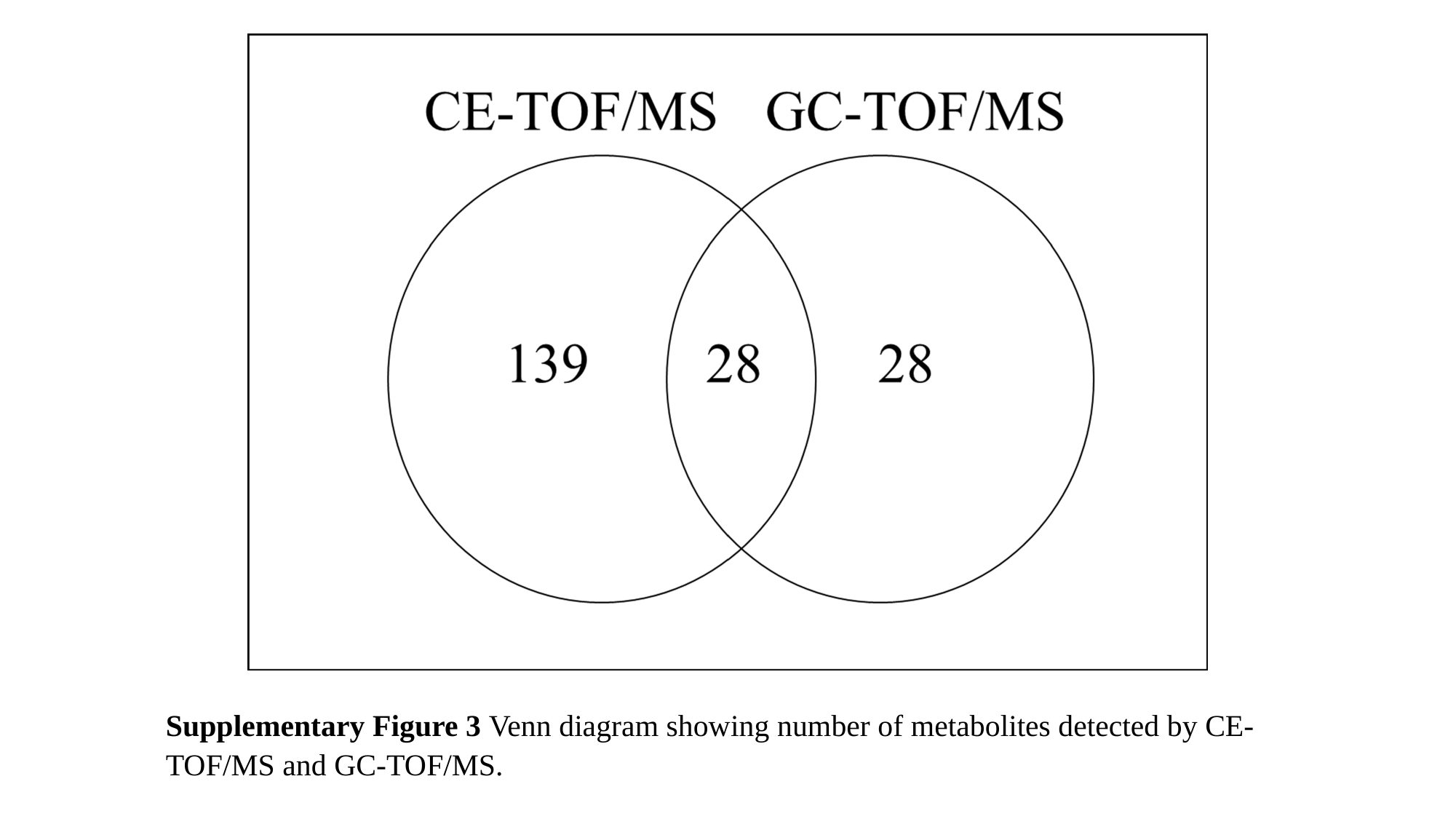

Supplementary Figure 3 Venn diagram showing number of metabolites detected by CE-TOF/MS and GC-TOF/MS.

## Slide 5
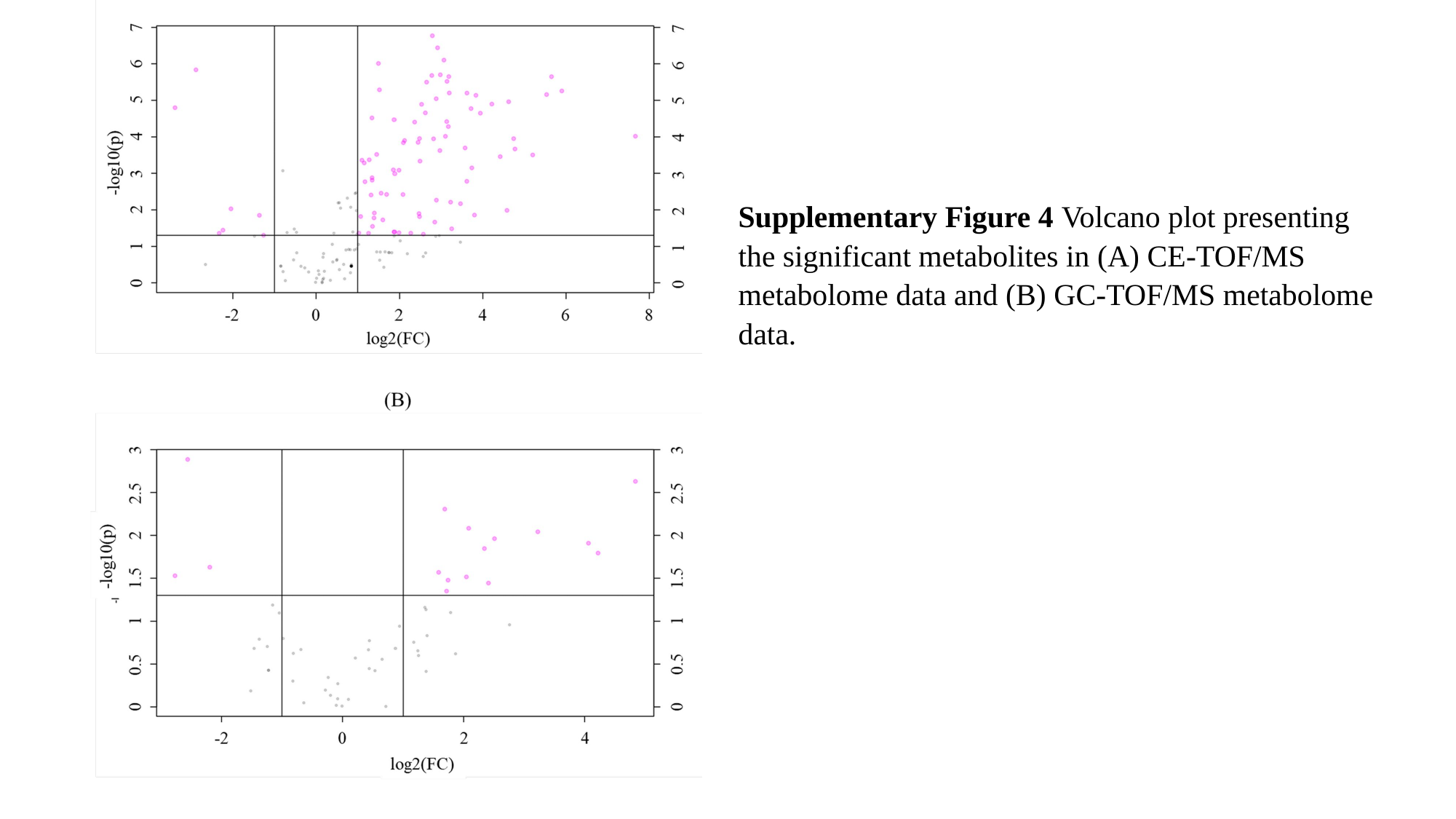

Supplementary Figure 4 Volcano plot presenting the significant metabolites in (A) CE-TOF/MS metabolome data and (B) GC-TOF/MS metabolome data.

## Slide 6
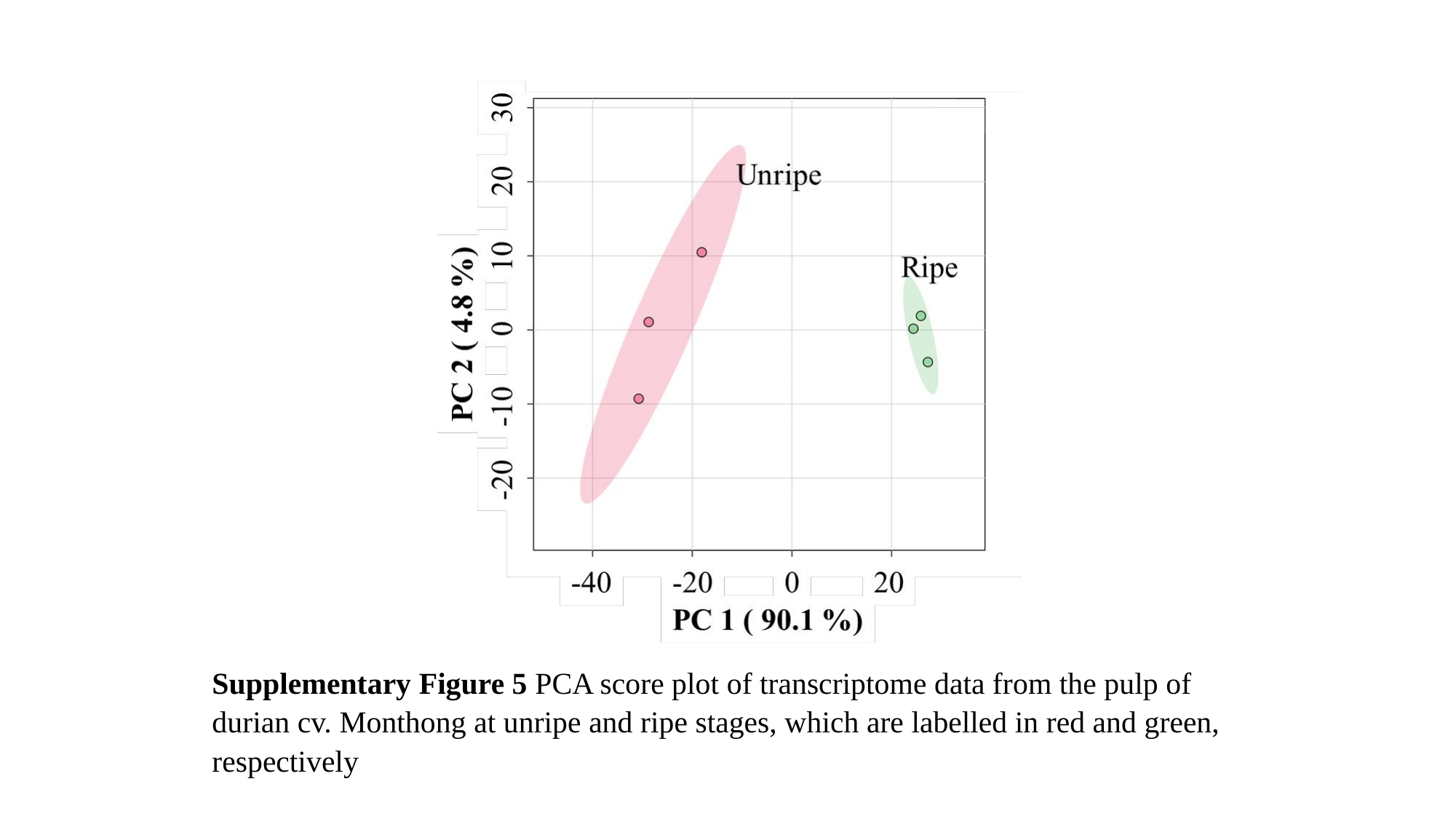

Supplementary Figure 5 PCA score plot of transcriptome data from the pulp of durian cv. Monthong at unripe and ripe stages, which are labelled in red and green, respectively

## Slide 7
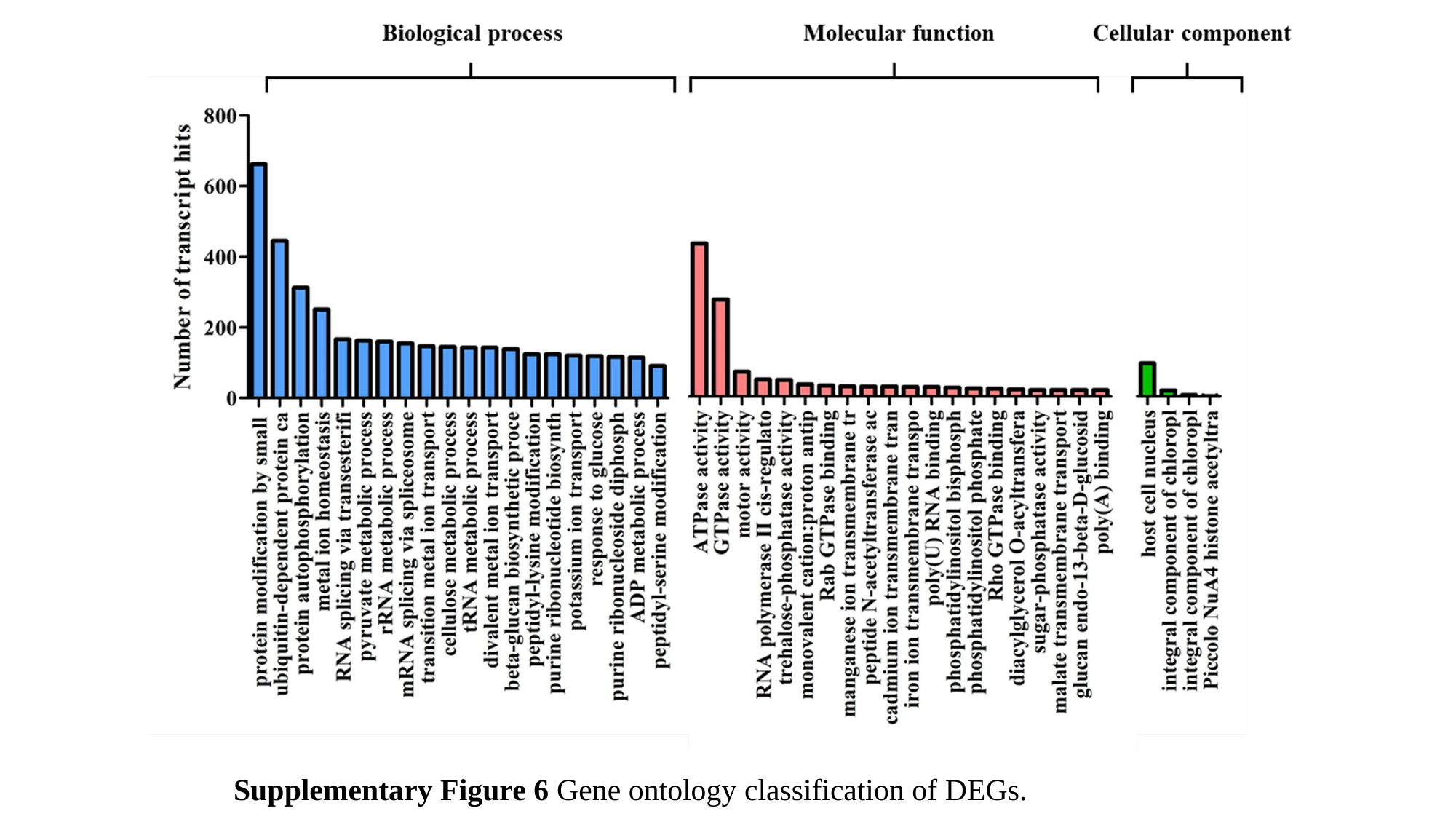

Supplementary Figure 6 Gene ontology classification of DEGs.

## Slide 8
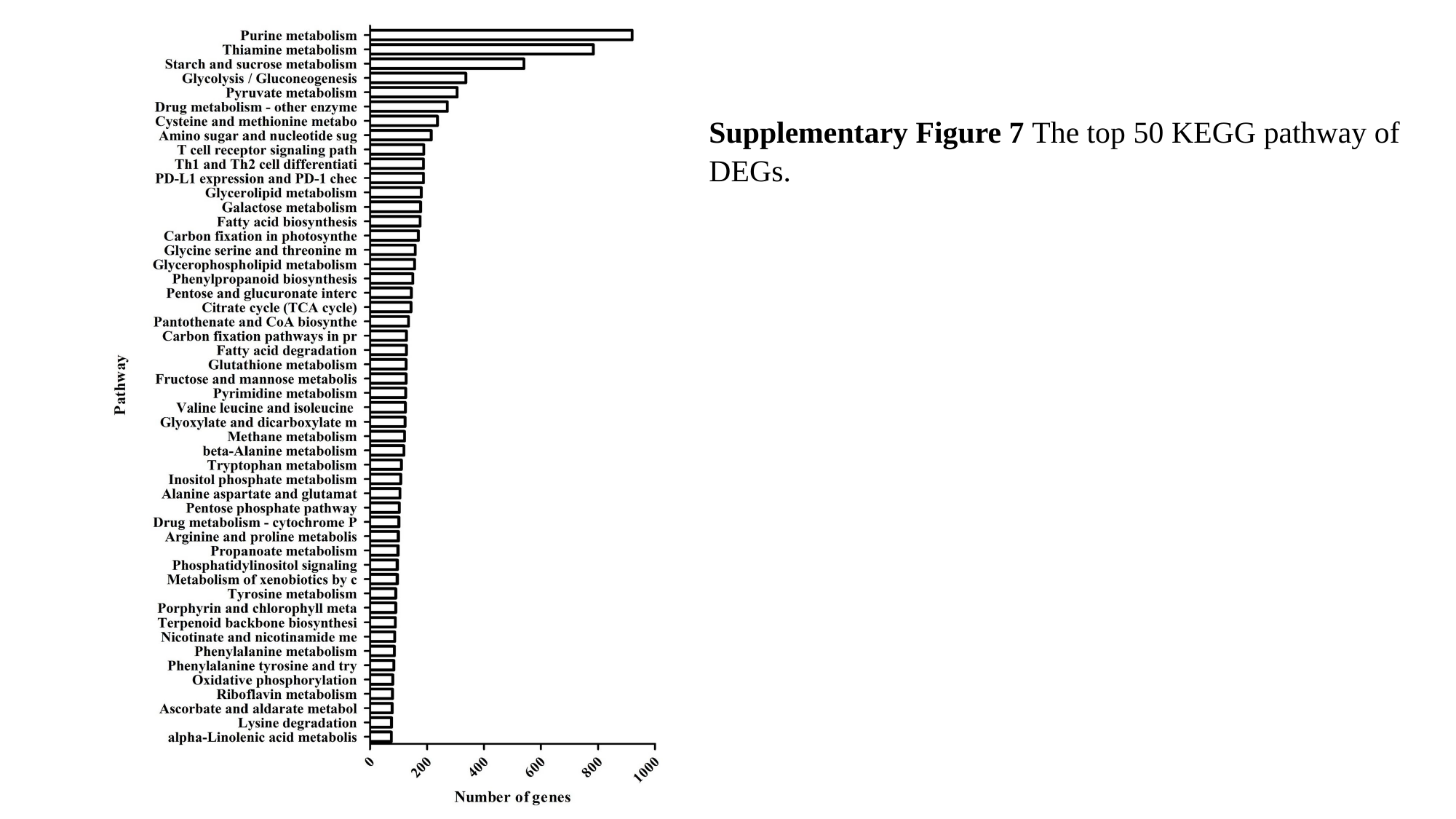

Supplementary Figure 7 The top 50 KEGG pathway of DEGs.
